# Supplementary material for: Clinical Genetic Testing for Hearing Loss: Implications for Genetic Counseling and Gene-Based Therapies
Source: Biomedicines. 2024 Jun 27;12(7):1427. doi: 10.3390/biomedicines12071427 (PMC11274279; doi:10.3390/biomedicines12071427)
Supplement: Supplementary file 1 [file biomedicines-12-01427-s001.zip › biomedicines-2912679-supplementary.pdf]

Supplementary Table S1. Summary of methods of Invitae and GeneDx hearing loss panel tests

Method	GeneDx	Invitae
Targeted capture	Proprietary next-generation sequencing with copy number variant (CNV) calling.	Hybridization-based at 250 depth including exons plus 20bp flanking introns. In-house algorithm for exonic deletions and duplications to determine CNVs at single exon resolution.
Gene-specific tests	• *GJB2, GJB6* – common recurrent deletions<br>• *TBX1* – sequencing, not deletion/duplication analysis<br>• *STRC, OTOA* – deletion/duplication analysis including multiplex ligation-dependent probe amplification (MLPA), not sequencing<br>• *ACTB, ESPN, TPRN* – whole gene deletion/duplication<br>• *PTPRQ* exons 4-6, *DSPP* exon 5, *TRIOBP* exon 7, *OTOA* exons 20-28 – sequence analysis not performed<br>• Mitochondrial genome – long-range PCR and sequencing for specific variants (MT-CO1: m.7445A>G; MT-RNR1:m. 1555 AsG; m.1494 C>T; MT-TL1:m.3243AsG; m.3291 T>C; MT-TS1: m.7511 T>C)	• *PMS2* exons 12-15 – alignment of *PMS2* and the *PMS2CL* pseudogene to align to *PMS2,* followed by confirmatory long-range amplification and PacBio SMRT sequencing or MLPA/MLPA-seq for CNVs<br>• *DSPP* – exon 5 not included<br>• *USH1C* – deletion/duplication analysis and exons 5-6 not included<br>• *COL11A1* -- deletion/duplication analysis and exons 16-17 not included; exon 57 not sequenced<br>• *OTOA* -- deletion/duplication analysis and sequencing of exons 20-28 not included<br>• *MSRB3* – exon 4 not sequenced<br>• Reported reduced sensitivity for indels >15bp, structural rearrangements (e.g. inversions, gene conversion events, translocations, etc.), variants embedded in sequence with complex architecture (e.g. short tandem repeats or segmental duplications), mosaicism, phasing, or mapping ambiguity; promoter, non-coding exons, and other non-coding regions are not covered
Confirmation tests	Information not available	Sanger sequencing, Pacific Biosciences SMRT sequencing, MLPA, MLPA-seg, Array CGH. Array CGH confirmation of NGS CNV calling performed by Invite Corporation.
Databases used	gnomAD, PROVEAN, dbSNP, ClinVar, Human Gene Mutation Database (HGMD)	ClinVar, HGMD, the Online Mendelian Inheritance in Man (OMIM)

Supplementary Table S2. Minor allele frequencies (MAF) and bioinformatics results for the 23 variants identified in six patients who underwent genetic testing for hearing loss.

Patient	Geno-type	Gene	cDNA variant	Amino acid variant	rsID	Novel Variant	AFR	AMR	ASJ	EAS	FIN	NFE	REM	SAS	DANN Rank Score	CADD PHRED	Bioinformatics Tools in dbnsfp42c with Deleterious Results
1	**Het[1]**	***MYO15A***	**c.7124_7127del**	**p.Asp2375Val fs*41**	**rs780170125**	**No**	**NA**	**NA**	**NA**	**NA**	**NA**	**NA**	**NA**	**NA**	**NA**	**NA**	**MT**
	**Het[1]**	***MYO15A***	**c.9109G>T**	**p.Glu3037***	**rs2046732267**	**No**	**0**	**0**	**0**	**0**	**0**	**0.000007**	**0**	**0**	**NA**	**44.0**	**MT**
	Het	*OTOG*	c.4856C>T	p.(Ser1619Leu)	rs867891665	--	0.0002	0.00002	0	0.00005	0.00002	0.00001	0.00005	0.00002	0.78	20.6	M-CAP,SIFT
	Het	*DMXL2*	c.7543A>G	p.(Met2515Val)	rs72729259	--	0.0005	0.0009	0.00003	0.0003	0.00002	0.0003	0.0002	0.0001	0.36	18.7	LRT,MT
2	**Hom**	***GJB2***	**c.35delG**	**p.Gly12Valfs*2**	**rs80338939**	**No**	**0.001**	**0.005**	**0.004**	**0.0002**	**0.0095**	**0.008**	**0.007**	**0.0006**	**NA**	**NA**	**MT**
	Het	*ALMS1*	c.11708G>A	p.(Arg3903Gln)	rs201673771	--	0.0001	0.0003	0.00003	0	0.0002	0.001	0.0005	0.00005	0.91	20.8	--
	Het	*ADGRV1*	c.13757A>G	p.(Glu4586Gly)	rs371917393	--	0	0.00007	0	0	0	0.00005	0.00006	0.00001	0.43	21.0	LRT,M-CAP,MT,PR,SIFT
	Het	*KARS1*	c.1259G>A	p.(Arg420Gln)	rs370244075	--	0.00005	0.0001	0	0.0001	0	0.00005	0.0005	0.00005	0.97	21.9	LRT,M-CAP,MT,PR
	Het	*MYO15A*	c.9620G>A	p.Arg3207His	rs199621031	--	0.00005	0.0008	0	0.00004	0	0.00002	0.0002	0	0.992	28.3	FATHMM,M-CAP,mLR, mSVM,MT,SIFT
3	**Het**	***MYO7A***	**c.2543G>A**	**p.(Arg848Gln)**	**rs1555082917**	**Yes**	**0**	**0**	**0**	**0**	**0**	**0.000004**	**0**	**0**	**0.999**	**32.0**	**FATHMM,MA,M-CAP, mLR,mSVM,MT,PR,SIFT**
	Het	*LRP2*	c.2426G>A	p.(Ser809Asn)	rs759522776	--	0	0	0	0	0	0.00002	0	0	0.41	19.6	FATHMM,LRT,MA, M-CAP,mLR,mSVM,MT
	Het	*ADGRV1*	c.12052G>A	p.(Val4018Ile)	NA	--	NA	NA	NA	NA	NA	NA	NA	NA	0.23	15.1	LRT,M-CAP
	Het	*OTOG*	c.7823delA	p.(Tyr2608Serfs*76)	NA	--	NA	NA	NA	NA	NA	NA	NA	NA	NA	NA	--
	Het	*PEX26*	c.98C>T	p.(Pro33Leu)	rs368118099	--	0.00004	0.00007	0.001	0	0	0.00009	0.0002	0	0.18	15.1	FATHMM,M-CAP,mLR
	Het	*BTD*[1]	c.1270G>C	p.(Asp424His)	rs13078881	--	0.007	0.02	0.03	0.0002	0.053	0.04	0.04	0.04	0.51	6.4	FATHMM,MA,mLR,mSVM, MT,PR,SIFT
4	**Het[1]**	***USH2A***	**c.2299delG**	**p.Glu767Serfs*21**	**rs80338903**	**No**	**0.0002**	**0.001**	**0**	**0**	**0**	**0.001**	**0.0007**	**0.00001**	**NA**	**NA**	**MT**
	**Het[1]**	***USH2A***	**c.13130C>A**	**p.Ser4377***	**rs111033385**	**No**	**0**	**0**	**0**	**0**	**0**	**0.000004**	**0**	**0**	**0.47**	**39.0**	**MT**
	Het	*COL4A4*	c.980A>G	p.(Glu327Gly)	rs375714304	--	0	0.00002	0	0	0	0.00004	0	0.00003	0.27	16.4	FATHMM,M-CAP, mLR,mSVM,MT
5	**Het**	***TMC1***	**c.928A>G**	**p.(Thr310Ala)**	**rs144501871**	**Yes**	**0.0002**	**0.00005**	**0**	**0**	**0**	**0**	**0.00006**	**0**	**0.27**	**12.6**	**--**
	Het	*SLC26A4*	c.1246A>C	p.Thr416Pro	rs28939086	--	0.00009	0.00008	0	0	0	0.0003	0.0001	0	0.83	30.0	FATHMM,LRT,MA,M-CAP, mLR,mSVM,MT,PR,SIFT
	Het	*VCAN*	c.3917C>G	p.(Ala1306Gly)	rs749496810	--	0	0	0	0	0	0	0	0.00001	0.37	14.5	FATHMM,MA,M-CAP,mLR
6	**Het**	***TECTA***	**c.2266A>G**	**p.(Lys756Glu)**	**rs141420954**	**Yes**	**0.00003**	**0.0003**	**0**	**0**	**0**	**0.0001**	**0.0003**	**0**	**0.25**	**21.1**	**LRT,SIFT**
	Het	*MITF*	c.560-7T>A	NA	rs200580325	--	0.0002	0.0001	0	0	0	0.0006	0.0004	0	--	18.25	MT

Abbreviations: AFR, African descent; AMR, admixed American; ASJ, Ashkenazi Jewish; CADD, Combined Annotation Dependent Depletion; DANN, deleterious annotation of genetic variants using neural networks (a rank score >0.5 is considered pathogenic); EAS, East Asian; FIN, Finnish; LRT, Likelihood Ratio Test; MA, MutationAssessor; mLR, MetaLR; mSVM, MetaSVM; MT, MutationTaster; NFE, non-Finnish European; PR, PROVEAN; REM, Remaining, SAS, South Asian. Variants *in bold* are likely causal of hearing loss in the six patients.

RefSeq NM#: *ADGRV1*, 032119; *ALMS1*, 015120; *BTD*, 0017281723; *COL4A4*, 000092; *DMXL2*, 001174116; *GJB2*, 004004; *KARS1*, 001130089; *LRP2*, 004525; *MITF*, 000248; *MYO7A*, 000260; *MYO15A*, 016239; *OTOG*, 001277269; *PEX26*, 017929; *SLC26A4*, 000441; *TECTA*, 005422; *TMC1*, 138691; *USH2A*, 206933; *VCAN*, 004385.

[1]Parental genotypes were not available to confirm compound heterozygosity. Given gnomAD MAFs, these variants are unlikely to be in linkage disequilibrium.

[2]This variant had the highest MAF of 0.043 within the Syrian Desert population in the Greater Middle East (GME) Variome database. This variant is likely benign and not causal of hearing loss.

Supplementary Table S3. Predicted effects of novel variants on protein structure or function

Gene	Variant	Protein Data Bank ID[a]	Affected Domain(s)[b]
MYO15A	p.(Glu3037*)	C7uduD	Loss of MyTH4 and FERM central domains which are required for stereocilia structure in hair cells [1]
MYO7A	p.(Arg848Gln)	C5mv9A	Variant lies within the third IQ motif, EF-hand binding site which contributes to the regulation of the motor action of the myosin protein [2]
TECTA	p.(Lys756Glu)	C8oesA	Variant lies within the von Willebrand factor, type D domain of the zonadhesin region, possibly affecting polypeptide assembly and the extracellular matrix of the tectorial membrane [3]
TMC1	p.(Thr310Ala)	C7uswB	Variant lies within the non-cytoplasmic domain or extracellular region of the transmembrane protein [4]; no motif identified with Prosite [5]

[a]Protein molecular modeling performed using Phyre2 [6]. [b]Domains identified using InterProScan 5 [7].

1. Moreland, Z.G.; Bird, J.E. Myosin Motors in Sensory Hair Bundle Assembly. *Curr Opin Cell Biol* **2022**, *79*, 102132, doi:10.1016/j.ceb.2022.102132.
2. Kuppa, A.; Sergeev, Y.V. Homology Modeling and Global Computational Mutagenesis of Human Myosin VIIa. *J Anal Pharm Res* **2021**, *10*, 41–48, doi:10.15406/japlr.2021.10.00364.
3. Hildebrand, M.S.; Morín, M.; Meyer, N.C.; Mayo, F.; Modamio-Hoybjor, S.; Mencía, A.; Olavarrieta, L.; Morales-Angulo, C.; Nishimura, C.J.; Workman, H.; et al. DFNA8/12 Caused by TECTA Mutations Is the Most Identified Subtype of Nonsyndromic Autosomal Dominant Hearing Loss. *Hum Mutat* **2011**, *32*, 825–834, doi:10.1002/humu.21512.
4. Santos, R.L.P.; Wajid, M.; Khan, M.N.; McArthur, N.; Pham, T.L.; Bhatti, A.; Lee, K.; Irshad, S.; Mir, A.; Yan, K.; et al. Novel Sequence Variants in the TMC1 Gene in Pakistani Families with Autosomal Recessive Hearing Impairment. *Hum Mutat* **2005**, *26*, 396, doi:10.1002/humu.9374.
5. Bairoch, A. PROSITE: A Dictionary of Sites and Patterns in Proteins. *Nucleic Acids Res* **1991**, *19 Suppl*, 2241–2245, doi:10.1093/nar/19.suppl.2241.
6. Bennett-Lovsey, R.M.; Herbert, A.D.; Sternberg, M.J.E.; Kelley, L.A. Exploring the Extremes of Sequence/Structure Space with Ensemble Fold Recognition in the Program Phyre. *Proteins* **2008**, *70*, 611–625, doi:10.1002/prot.21688.
7. Quevillon, E.; Silventoinen, V.; Pillai, S.; Harte, N.; Mulder, N.; Apweiler, R.; Lopez, R. InterProScan: Protein Domains Identifier. *Nucleic Acids Res* **2005**, *33*, W116-120, doi:10.1093/nar/gki442.
